# AEBP1 down regulation induced cell death pathway depends on PTEN status of glioma cells

**DOI:** 10.1038/s41598-019-51068-1

**Published:** 2019-10-10

**Authors:** Swati Sinha, Arun Renganathan, Prathima B. Nagendra, Vasudeva Bhat, Brian Steve Mathew, Manchanahalli R. Satyanarayana Rao

**Affiliations:** 1 Molecular Biology and Genetics Unit, Jawaharlal Nehru Centre for Advance Scientific Research, Bangalore, Karnataka 560064 India; 2 Department of Surgery, Washington University in St. Louis, St. Louis, MO USA; 3 Gynaecology Oncology Group, School of Biomedical Sciences and Pharmacy, University of Newcastle, Callaghan, New South Wales Australia; 4 Department of Immunology, Faculty of Health Sciences, University of Manitoba, Winnipeg, Manitoba Canada

**Keywords:** CNS cancer, Cancer

## Abstract

Glioblastoma (GBM) is the most common aggressive form of brain cancer with overall dismal prognosis (10–12 months) despite all current multimodal treatments. Previously we identified adipocyte enhancer binding protein 1 (AEBP1) as a differentially regulated gene in GBM. On probing the role of AEBP1 over expression in glioblastoma, we found that both cellular proliferation and survival were affected upon AEBP1 silencing in glioma cells, resulting in cell death. In the present study we report that the classical caspase pathway components are not activated in cell death induced by AEBP1 down regulation in PTEN-deficient (U87MG and U138MG) cells. PARP-1 was not cleaved but over-activated under AEBP1 down regulation which leads to the synthesis of PAR in the nucleus triggering the release of AIF from the mitochondria. Subsequently, AIF translocates to the nucleus along with MIF causing chromatinolysis. AEBP1 positively regulates PI3KinaseCβ by the binding to AE-1 binding element in the PI3KinaseCβ promoter. Loss of PI3KinaseCβ expression under AEBP1 depleted condition leads to excessive DNA damage and activation of PARP-1. Furthermore, over expression of PIK3CB (in trans) in U138MG cells prevents DNA damage in these AEBP1 depleted cells. On the contrary, AEBP1 down regulation induces caspase-dependent cell death in PTEN-proficient (LN18 and LN229) cells. Ectopic expression of wild-type PTEN in PTEN-deficient U138MG cells results in the activation of canonical caspase and Akt dependent cell death. Collectively, our findings define AEBP1 as a potential oncogenic driver in glioma, with potential implications for therapeutic intervention.

## Introduction

Glioblastoma multiforme (GBM) is a very aggressive form of brain cancer with very poor prognosis. These cancer patients have overall survival span averaging one-year despite of multimodality therapeutic interventions involving surgery, radiotherapy with concomitant adjuvant chemotherapy. A better understanding of the genetic and the epigenetic determinants of GBM are being pursued actively for identifying novel effective therapeutic targets to treat these aggressive tumor cells. We had observed earlier that adipocyte enhancer binding protein 1 (AEBP1) expression to be up-regulated in primary GBMs as opposed to progressive secondary GBMs through transcriptome analysis^1^. AEBP1 was originally discovered as a transcriptional repressor that binds to the AE-1 element of the ap-2 gene which codes for the fatty acid binding protein 4 (FABP4)^2^. Fabp4 is involved in the differentiation of preadipocytes to mature adipocytes and smooth muscle cell differentiation^3^. In addition to its role in the adipose tissue, AEBP1 is being increasingly implicated in different cancers. AEBP1 expression is up-regulated in prostate cancer^4^ and its up-regulation confers acquired resistance to BRAF (V600E) inhibition in melanoma^5^. AEBP1 is also reported to promote mammary cell hyperplasia through up-regulation of *Hedgehog* and NFkB pathway components^6^. A group of 10 genes including AEBP1 is linked to high metastasis and poor prognosis in serous ovarian cancer^7^. In an initial effort to understand the role of AEBP1 in primary glioma, we performed global gene expression profiling in AEBP1 down regulated U87MG glioma cell line and identified a large number of perturbed genes belonging to categories of cell cycle, differentiation, proliferation and apoptosis^8^. We also showed that down regulation of AEBP1 resulted in cell death of both U87MG and U138MG cells suggesting that AEBP1 may play an essential role as an oncogenic protein. This assumes great importance considering the fact that migrating GBM cells are resistant to conventional apoptosis (Type I programmed cell death) due to the over expression of IAPs^9^, and therefore to radiotherapy and conventional chemotherapy^10^, as a consequence of which GBM (Grade IV) patients have a poor prognosis with a median survival of only14.6 months^11^.

The conventional mechanisms of cell death are apoptosis, autophagy, and necroptosis. Although apoptosis is generally characterized by nuclear pyknosis, chromatin condensation, and phosphatidyl serine exposure on the plasma membrane, these are not truly specific biomarkers for caspase activation. In an alternative, caspase-independent pathway, phylogenetically conserved death effector molecule termed AIF has been shown to mediate chromatin condensation and induce phosphatidyl serine exposure when caspase activation is inhibited^12,13^. In some paradigms of yeast cell death^14^ and in mammalian neurons^15^, AIF is necessary for cell death induction. AIF is typically confined to mitochondria but translocates to the nucleus under the influence of poly (ADP-ribose) (PAR) polymerase-1 (PARP-1) activation when cell death is induced^16,17^. This distinct cell death pathway mediated by events such as over activation of PARP1, PAR synthesis, nuclear AIF translocation and large scale DNA fragmentation are specific to the phenomenon of parthanatos^18,19^. This unique parthanatos distinguishes itself from caspase dependent apoptosis pathway in not involving relevant caspases. Our previous study shows that down regulation of AEBP1 in glioma cells resulted in cell death^8^, thus we were interested in exploring the actual mechanism of cell death triggered by depletion of AEBP1. In the present study, we deciphered that AEBP1 depletion-induced cell death mechanism in glioma cells and its dependence on the genetic background of tumor cells. We demonstrate that AEBP1 down regulation in Phosphatase and tensin homolog (PTEN)-deficient (U87MG and U138MG) cells causes phosphatidylinositol-4,5-bisphosphate 3-kinase catalytic subunit beta (PIK3CB) depletion by directly decreasing its transcript levels leading to large-scale DNA damage, hyperactivation of PARP-1, PAR polymer mediated release of AIF from mitochondria and subsequent caspase-independent cell death by Parthanatos^20^. On the other hand, AEBP1 down regulation in PTEN-proficient (LN18 and LN229) cells induces the classical caspase-dependent cell death pathway. It has been previously established that the lipid kinase activity of PI3KCβ is essential to maintain PI3Kinase signaling in PTEN deficient cells. Also PI3Kinase is essential for the maintenance of genomic integrity^21^. Furthermore, ectopic expression of PTEN wild-type cDNA in U138MG cells (PTEN deficient) induced caspase-dependent cell death pathway in AEBP1 depleted cells. Thus, PI3kinaseβ assumes importance in PTEN deficient tumors like glioma as its ablation impedes tumorigenesis. This is the first report of a transcription factor (AEBP1) acting as a potential oncogenic protein in GBM by regulating the expression of PI3KCB, which is increasingly being recognized as an important molecule in the pathobiology of many cancers^22^.

## Materials and Methods

### Cell culture and reagents

Glioma cells, U87MG, U138MG, LN18 and LN229 were purchased from ATCC and cultured in DMEM (Sigma Aldrich) supplemented with 10% FBS (Gibco) and 1% penicillin/streptomycin (Gibco) at 37 °C with 5% CO_2_. All fine chemicals were purchased from Sigma Aldrich and Life Technologies unless otherwise specified.

### AEBP1 silencing

For down regulation of AEBP1 with small interfering RNAs (siRNA), GBM cells were transfected with 100 nM of siRNA pool (Supplementary Table S2, Dharmacon Inc.,USA) targeted against AEBP1 or non-targeting scrambled siRNA pool by using Lipofectamine 2000 (Life technologies, USA). In our earlier study we observed no significant loss of cell viability in U138MG and U87MG cells during the first 4 days of post transfection even though AEBP1 was significantly down regulated at this time period. Furthermore, the reduction in cell viability was observed from 5^th^ day onwards which suggested us that silencing of AEBP1 resulted in the loss of proliferative potential or cell death^8^. Nevertheless, we assessed the effect of AEBP1 silencing in U138MG cells on selected on-target (CAMTA1, MDM2, UBE3A, ARNT, CD93 and MAPK13) and off target genes (TP53INP1, DMTF1, ERBB2P and SIVA1) expression levels. These genes were categorized as on-target and off-target genes with respect to the presence of AE 1 binding element in their promoters^8^. As shown in the Supplementary Fig. 1 AEBP1 silencing in U138MG does perturb its on-target genes but not its off-target genes. Thus, in this study siRNA pool was replenished every 60 hours in case of U87MG, LN18 and LN299 cells and every 36 hours in the case of U138MG cell line for over a period of 9 days to maintain the Aebp1 depleted condition. Total RNA was extracted using Trizol reagent and reverse transcription was carried out with RevertAid first strand cDNA synthesis kit (Thermo Scientific, USA) according to the manufacturer’s protocol.

### Antibodies

Antibodies used in this study are as follows: rabbit monoclonal anti-cleaved caspase 8 antibody (Novus Biologicals), rabbit monoclonal anti-cleaved caspase 9 antibody (Novus Biologicals), rabbit monoclonal anti-cleaved caspase 3 antibody (Novus Biologicals), rabbit polyclonal anti-cleaved PARP-1 antibody (Abcam), rabbit polyclonal anti-Bid cleavage site antibody (Abcam), rabbit monoclonal anti-AIF antibody (Abcam), mouse monoclonal anti-PAR antibody (Abcam), mouse monoclonal anti-MIF antibody (Cusabio), rabbit monoclonal anti-PI3Kinase p110 beta antibody (Abcam), mouse monoclonal anti-γH2AX antibody (raised in house) and rabbit polyclonal anti-GAPDH antibody (Abcam).

### Western blot analysis

Proteins were separated by 10–15% SDS-PAGE, transferred to nitrocellulose membranes (Amersham Bioscience), blocked with 5% skimmed milk/PBST, and then probed with antibodies as indicated. Protein bands were detected by chemiluminescence with the ECL system (Pierce) according to the manufacture’s protocol.

### Luciferase reporter assay for promoter activity of PI3kinase catalytic sub unit beta

PI3kinase 110 CB promoter (−2000–+200 bp) was amplified from genomic DNA isolated from U138MG glioma cells and cloned into the *XhoI* and *HindIII* sites of the basic pGL3-promoter vector (Promega Corp. USA). 2.5 × 10^4^ cells were seeded per well in a 24-well plate, 24 hours prior to transfection. 2 μg of various reporter constructs were co-transfected in U138MG and HeLa cells along with 200 ng of pCMVβ, (internal control plasmid pGL3-β Gal that expresses the β-galactosidase gene under the control of CMV promoter) as a transfection control. After 24 hours of post transfection, cells were subjected to AEBP1 silencing for different time periods. Cells were processed as prescribed in Pierce Firefly Luciferase Glow Assay Kit protocol (Thermoscientific USA) and relative light units were measured in a Luminometer (Berthold detection systems). β-galactosidase activity was measured by fluorometric assay and used to normalize transfection efficiency. The primers used in the study are listed in Supplementary Table 2.

### Caspase-Glo 3/7 assay

U138MG cells were transfected with 100 nM of siRNA pool targeted against AEBP1 and cultured over a period of 11 days. Caspase activity was measured at different time points by using the Caspase-Glo^TM^3/7 assay kit (Promega, WI) according to the manufacturer’s protocol. Briefly, Caspase-Glo 3/7 solution was added to the culture media and incubated at 37 °C for 30 min in a cell culture incubator. The contents of each well were mixed carefully with a micropipette and 50 µl of this mixture was transferred to a 96-well polystyrene assay plate (Corning Inc., NY) and the light emitted was read in a microplate luminometer at 570 nm.

### Pan-caspase inhibition and cell viability assay

U138MG cells were plated in 96-well plates and were transfected every 36 hours with 100 nM of siRNA pool designed against AEBP1 or non-targeting scrambled siRNA as a control for a period of 9 days. HeLa cells were treated with 10 µg/ml doxycycline (Sigma Aldrich) for inducing caspase mediated apoptosis. To check the involvement of classical caspase pathway, pan-caspase Inhibitor Z-VAD-FMK (Promega) was added at a final concentration of 20 mM, two hours prior to either doxycycline treatment or AEBP1 siRNA treatment. After completion of treatment, cells were treated with MTT (3-[4-5 dimethyl thaizole-2-yl]2-5 diphenyltetrazolium bromide (Sigma-Aldrich) for 4 hours and the formazan crystals formed by metabolically active cells was solubilized in DMSO and measured in a spectrophotometer at 550 nm.

### Immunofluorescence assay

Cells were grown on coverslips and subjected to AEBP1 down regulation as described above. After desired period of incubation, cells were fixed with 4% paraformaldehyde for 20 minutes, permeabilized with 0.1% triton X-100 for 10 minutes and blocked with 1% BSA. Cells were incubated with primary antibodies in 1X PBS with 1% BSA at 4 °C for overnight. The primary antibodies used were rabbit anti-AIF antibody (Abcam), rabbit anti- PI3 Kinase p110 beta antibody (Abcam) and mouse monoclonal anti-γH2AX antibody (raised in house). Appropriate secondary antibodies conjugated with Alexa fluor 488, 568, or 633 (Invitrogen/Molecular Probes) were used. The secondary antibody was added and incubated for one hour at room temperature. Mitotracker Red CMXRos dye (Life Technologies) was used to assess the mitochondrial outer membrane potential (MOMP).

### Immunoprecipitation

Cells were treated with lysis buffer (50 mM Tris pH7.4, 0.1% NP40, 100 mM NaCl, 0.1 mM EDTA, 1% Glycerol, 1 nM PMSF, 1X Protease Inhibitor Complex and 5 mM MgCl_2_).The total cell lysate was precleared with Protein A agarose beads (Invitrogen) and the lysate was incubated with antibody of interest, overnight at 4 °C in an end to end rotator. Preimmune-IgG was used as a negative control. Protein-A-agarose beads were added in 1/10^th^ measures of the lysate at 4 °C for 4 hours. Beads were collected and washed with the lysis buffer 2–3 times. These samples were prepared to load on a 10–15% SDS-PAGE and were blotted on to a membrane and probed.

### Chromatin immunoprecipitation (ChIP)

Cells were cross-linked with 1% formaldehyde and the reaction was quenched with glycine (0.125 M). They were resuspended in ChIP incubation buffer (0.1% SDS, 0.5% Triton X-100, 20mMTris-HCl pH8, and 150 mM NaCl) and sheared using a bioruptor sonicator (Diagenode). Sonication efficiency was standardized to get an enrichment of DNA fragments in the range of 600 bp to 2 kb. The sonicated chromatin was centrifuged for 15 min and resuspended in IP buffer (20 mM Tris/HCl pH7.4, 150 mM NaCl, 2 mM EDTA, 10% glycerol, 1% Triton X-100 and Mammalian Protease Inhibitor Complex) and the resultant lysate was precleared using Protein A agarose beads (Invitrogen) and then incubated with 3 μg of histone H3 antibody or 3 μg of mouse IgG (negative control) for 12 h at 4 °C. The immune complex was captured with Protein A agarose beads for 4 h at 4 °C. The beads were successively washed twice with Buffer 1 (0.1% SDS, 0.1% deoxycholate, 1% Triton-X 100, 0.15 M NaCl, 1 mM EDTA, 0.5 mM EGTA, 20 mM HEPES [pH 7.6]) and once with Buffer 2 (0.1% SDS, 0.1% sodium deoxycholate, 1% Triton-X 100, 0.5 M NaCl, 1 mM EDTA, 0.5 mM EGTA, 20 mM HEPES [pH 7.6]). The captured chromatin fragments were eluted by incubation of the beads with elution buffer (1% SDS, 0.1 M NaHCO_3_) at room temperature for 20 min. The proteins associated with precipitated chromatin was further resolved on 10% SDS PAGE, blotted onto a membrane and probed with AIF and γH2AX antibodies. For ChIP PCR the DNA associated with precipitated chromatin was isolated and analysed using qPCR. The primers used in the study are listed in Table S2.

### Subcellular fractionation

Cells were trypsinised and washed with PBS and lysed with Buffer 1-Digitonin Buffer (150 mM NaCl, 50 mM HEPES, 7.5 µg/ml digitonin +1% mammalian protease inhibitor Complex) in an end to end rotator for 10 minutes at 4 °C and centrifuged at 2000 × g. The supernatant was taken to be the cytosolic fraction. The resultant pellet was resuspended in Buffer 2-NP40 Buffer (150 mM NaCl, 50 mM HEPES pH 7.4, 1% NP40), left on ice for half an hour and centrifuged at 7000 × g. The supernatant contained the membrane rich fractions. The pellet was further resuspended in ice cold Buffer 3- RIPA buffer (150 mM NaCl, 50 mM HEPES, 0.5% sodium deoxycholate, 0.1% SDS and 1% DNase) and incubated in an end to end rotator for an hour at 4 °C after which the suspension was centrifuged at 7000 × g for 10 minutes. The pellet containing insoluble proteins was resuspended in the RIPA extraction buffer- Buffer 4 (150 mM NaCl, 50 mM HEPES, 0.5% sodium deoxycholate, 1% SDS, 100 mM dithiothreitol), and the resultant suspension was mixed with the supernatant from buffer 3 to obtain the nuclear lysate. The purity of the fractions was confirmed using western blot probing with fraction specific protein marker antibodies.

### Flow cytometry

Cells were grown in 65 mm dishes and subjected to AEBP1 down regulation up to 9 days, as described earlier. Cells were harvested at different time points to analyse the change in Mitochondrial Outer Membrane Potential, using the MitoTracker® Red CMXRos (Life Technologies) (100 nM) as per manufacturer’s protocol. Briefly, after the treatment cells were trypsinised and centrifuged at 900 × g and washed with PBS. The cell pellet was resuspended in PBS. Un-transfected cells without adding the mitotracker dye were used as template to adjust the frequency and voltage to mask the autofluorescence. MitoTracker® Red CMXRos dye was added to scrambled siRNA and AEBP1 siRNA treated samples. Cells were analyzed using FACS Aria Cell Sorter (BD Biosciences) with an emission wavelength of 577 nm.

### Analysis of DNA fragmentation

After the AEBP1 siRNA treatment as already mentioned above, cells were trypsinised and centrifuged at 900 × g and washed with PBS and fixed for half an hour in 50% methanol at 4 °C. The solution was diluted in the ratio 1:1 with PBS and the cells were centrifuged down at 900 × g. This was followed by RNase treatment (50 µg/ml) for 1–3 hours at 37 °C and incubated with PI for 1 hour at 37 °C. Cells were analyzed using FACS Aria Cell Sorter (BD Biosciences) with an emission wavelength range of 580–630 nm.

### Intra-nucleosomal DNA ladder formation assay

Cells were collected after AEBP1 siRNA treatment and DNA fragments were isolated according to the standard protocol for DNA isolation^23^. Briefly, glioma cells were treated with AEBP1 siRNA, harvested, counted and washed with PBS at 4 °C. The cells were pelleted by centrifugation at 200 g at 4 °C. The pellet was suspended in DNA lysis buffer (1 M Tris, pH 8.0, 0.5 M EDTA and 75% sodium lauryl sarcosine) and incubated overnight with proteinase K (0.5 mg/mL) at 50 °C. After overnight incubation, RNase was added and the cells were again incubated for 1 h at 50 °C. DNA was extracted using phenol/chloroform (1:1 v/v) and then electrophoresed in 2% agarose gel for 2 h at 50 V. The gel was stained with ethidium bromide (0.5 μg/mL) and photographed under UV trans-illuminator.

### Quantitative real time-PCR measurement of AEBP1 and PI3KCB genes

To assess the gene expression of AEBP1 and PI3KCB upon siAEBP1, qRT-PCR were performed from total RNA extracted from experimental cells by using Trizol reagent and reverse transcription was carried out with RevertAid first strandcDNA synthesis kit (ThermoScientific, USA) according to the manufacturer’s protocol. qRT-PCR was performed in BioRad CFX96 Real-Time PCR System by using SensiFAST SYBR® No-ROX Kit (Bioline Reagents Ltd). Each qRT-PCR reaction (in 25 μL) involved 12.5 μL 2x SensiFAST SYBR No-ROX Mix, 1 μL of each primer, 2 μL cDNA and 8.5 μL H2O. The cycling conditions included an initial single cycle (95 °C for 3 min), and followed by 40 cycles (95 °C for 15 s; 57–60 °C for 15 s; 72 °C for 20 s). All amplifications were followed by dissociation curve analysis of the amplified products. Specific primers were designed using the NCBI, specificities were confirmed with BLAST and gene expression levels were normalized with GAPDH to attain the relative expression by using 2^(−ΔΔCt)^ value methods (Supplementary Table S2).

### Over expression of PI3KCB and ectopic expression of PTEN^WT^ in U138MG cells

The PTEN wild type cDNA was amplified from LN18 cells derived cDNA by using sequence specific primers that incorporated 5′ *HindIII* and 3′ *BamHI* restriction sites (Supplementary Table S2) which was subsequently cloned into the plasmid vector pcDNA3.1(+) vector to generate pcDNA-PTEN^WT^. PIK3CB ORF clone from GenScript (OHu21708D) was procured to over express PIK3C beta in U138MG cells. Briefly, U138MG cells were transfected with pcDNA-PIK3CB or pcDNA-PTEN^WT^ construct or pcDNA3.1^+^ (negative control) by using Lipofectamine 2000 (Life technologies). Over expression of PIK3C beta and ectopic expression of PTEN^WT^ was confirmed by probing PIK3Cbeta or PTEN in total protein extracts after 72 of post-transfection with anti-PIK3C beta or anti-PTEN antibody.

### Statistical analysis

All statistical data were analysed by GraphPad Prism 5 software. Two-tailed Student’s t-test and two-way ANOVA tests were used appropriately, and a P < 0.05 was considered statistically significant. Unpaired parametric t-tests were used to analyze means of two groups. Bar graphs show the mean ± SD of biological replicates.

## Results

### Caspases are not activated under AEBP1 down regulated condition in U138MG cells

**Figure 1:**
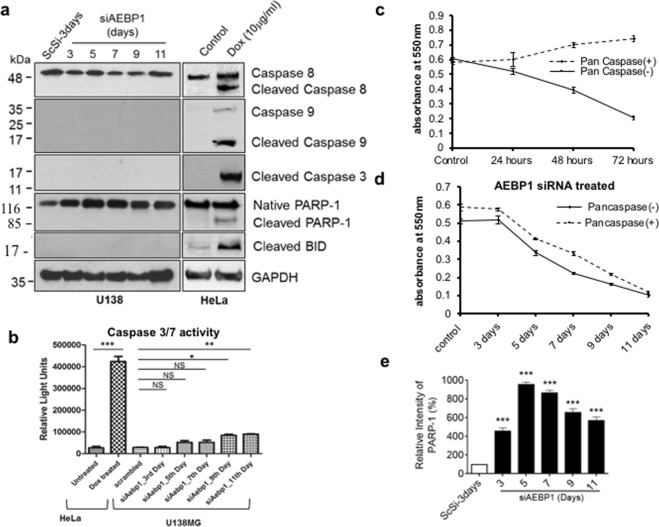
Caspases are not activated upon cell death induced by AEBP1 down regulation in U138MG cells. (**a**) Western blots of cleaved caspases 8, 9 and 3, cleaved Bid, native PARP-1 and cleaved PARP-1 with GAPDH as the loading control. Cells were treated with siAEBP1 upto 11 days and harvested at different time points as mentioned. Scrambled siRNA treated U138MG and untreated HeLa cells were used as negative controls while doxycycline (10 μg/ml) treated HeLa cells served as a positive control. (**b**) Caspase Glo Assay, probed for Caspase 3/7 in U138MG cells upon AEBP1 down regulation. Negative controls were scrambled siRNA treated U138MG and untreated HeLa cells while doxycycline (10 μg/ml) treated HeLa cells served as positive control. *p < 0.05, **p < 0.001, ***p < 0.0001 and NS, Non-significant. (Untreated versus Dox treated in HeLa cells and Scrambled (ScSi) versus siAEBP1 in U138MG cells). One-way ANOVA followed by Dunnett’s test was used to evaluate the statistical significance. The data are expressed as mean ± SD (n = 3). (**c**) Activation of caspase pathway in U138MG cells upon doxycycline treatment using MTT assay. Pan-caspase inhibitors treatment shows restoration of cellular viability. (**d)** AEBP1 down regulation induces cell death in U138MG cells despite addition of Pan Caspase inhibitors. Two sets of cells were treated with AEBP1 siRNA while one of them was treated with Pan-caspase inhibitors, 2 hours post siRNA treatment. Cellular viability was measured using the MTT assay. (**e**) Densitometry analysis of uncleaved PARP-1 upon down regulation of AEBP1 (Fig. 1a); PARP-1 is maximally over expressed 5 days post treatment. ***p < 0.0001, scrambled (ScSi) versus siAEBP1 treated cells. One-way ANOVA followed by Dunnett’s test was used to evaluate the statistical significance. The data are expressed as mean ± SD (n = 3).

In order to understand the mechanism of cell death induced upon AEBP1 siRNA mediated down regulation in U138MG glioma cells, we initially explored the involvement of classical caspase mediated apoptotic pathway in this process^24^. On probing the initiator caspases, both caspase 8 (extrinsic or ligand mediated pathway) and caspase 9 (intrinsic or mitochondrial pathway), we observed that neither of these caspases were activated upon AEBP1 down regulation in U138MG glioma cells (Fig. 1a). In the doxycycline treated HeLa cells which was used as a positive control^25,26^, we observed the activation of caspase 8, 9 and 3. Further, we also probed the mitochondrial membrane protein Bid as Bid cleavage has been reported to activate caspase 8 through apoptosome complex^27^. Here also we did not observe any cleaved Bid product in AEBP1 down regulated U138MG cells. Next we investigated the cleaved caspase 3, which is a common downstream signaling event in the caspase cascade and we did not observe cleaved caspase 3 under these conditions (Fig. 1a). These findings were further validated by probing for both the executioner caspases, caspase 3 and 7 using caspase glo3/7 assay using doxycycline treated HeLa cells as a positive control. While doxycycline activated caspases 3/7 were observed in HeLa cells as seen in Fig. 1b, a similar activation was not observed in AEBP1 down regulated U138MG cells. To further substantiate the observation that caspase pathway is not involved in AEBP1 down regulated cell death of U138MG cells, we carried out cell viability assay of these AEBP1 depleted U138MG cells in the presence and absence of pan caspase inhibitor Z-VAD-FMK, which inhibits caspase dependent cell death. For this purpose, AEBP1 was silenced in U138MG glioma cells using 100 nM AEBP1 siRNA pool, which was replenished every 36 hours over a period of 9 days and assessed for cellular viability MTT (3-(4,5-Dimethylthiazol-2-yl)-2,5-Diphenyltetrazolium Bromide)assay. MTT assay is a colorimetric assay for assessing cell metabolic activity and consequently cell viability and proliferation^28^. NAD(P)H-dependent cellular oxidoreductase enzymes reduce MTT to insoluble, purple formazan crystals which was further solubilised in solvent. Absorbance of the solution was quantified by measuring at 500 and 600 nm by a spectrophotometer. In parallel, cells were treated with doxycycline, an inducer of caspase mediated cell death. Pan-caspase inhibitor was added 12 hours after each treatment and subjected to MTT assay as mentioned above. We observed that upon addition of pan caspase inhibitor, doxycycline treated U138MG cells showed no loss in cell viability (Fig. 1c), whereas cell death was observed in AEBP1 down regulated U138MG cells (Fig. 1d). All these results clearly suggest that classical caspase pathway components are indeed present in U138MG glioma cells, but however, the pathway is not activated under AEBP1 depleted conditions. Finally, we probed for the cleavage of DNA nick sensing repair enzyme, PARP-1. PARP-1 is cleaved and inactivated by cleaved caspase 3. As expected we did not observe any cleaved PARP-1 under the experimental conditions, instead we noted higher level expression of native PARP-1 in comparison to scrambled siRNA (ScSi) treated U138MG cells (Fig. 1e). However, under similar experimental conditions, doxycycline treated HeLa cells did show cleaved PARP-1 (Fig. 1a).

### DNA damage and PARP-1 over activation in AEBP1 depletion condition

**Figure 2:**
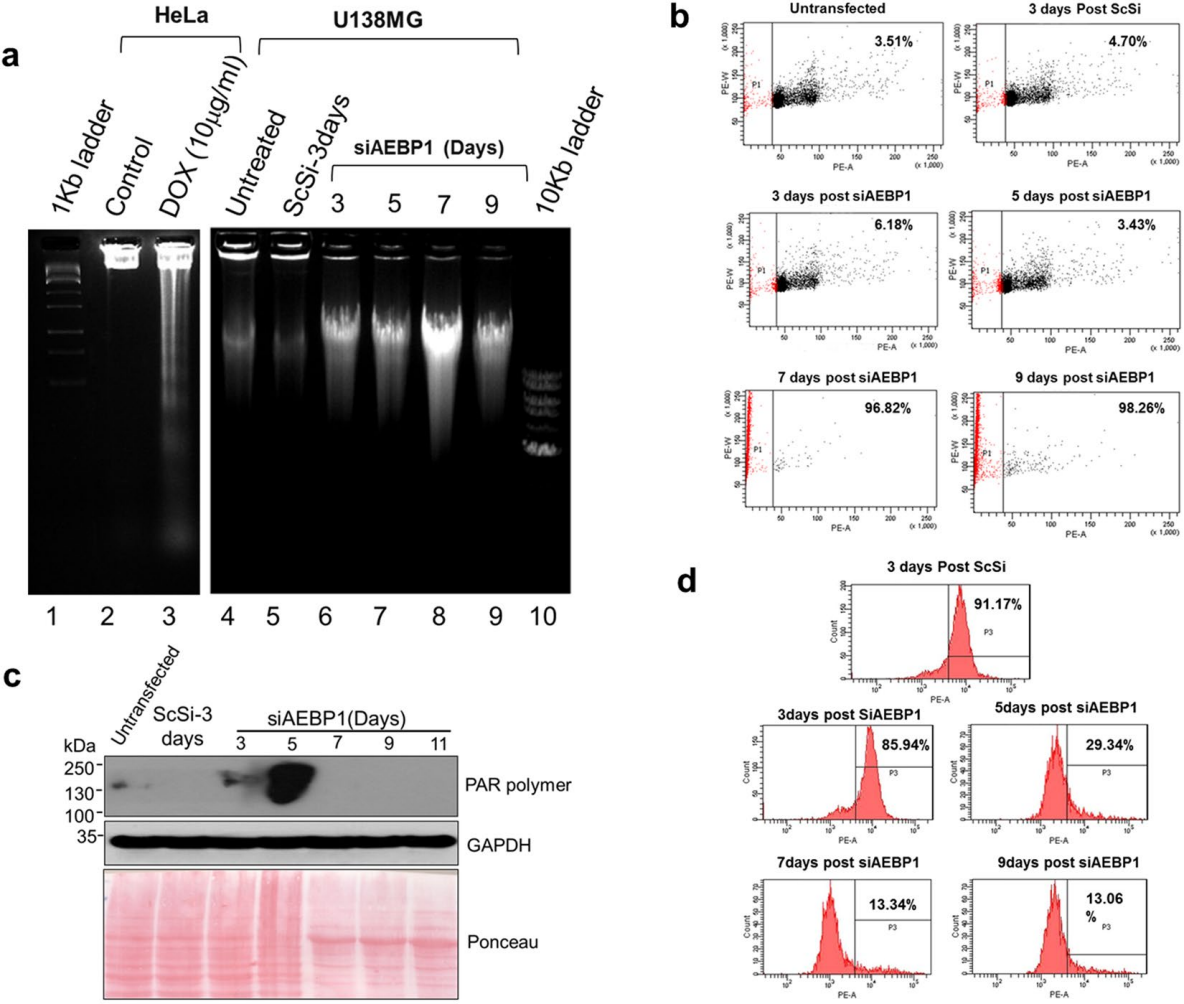
Activated PARP-1 leads to loss of MOMP mediated chromatinolysis. (**a**) AEBP1 silenced U138MG glioma cells (Lanes 6–9) show accumulation of large 50Kb fragments while doxycycline treated HeLa cells (Lane 3) show typical nucleosomal ladder pattern characteristic of apoptosis. (**b**) FACS analysis shows accumulation of sub n population of cells in AEBP1 down-regulated U138MG cells. AEBP1 siRNA treated cells were labeled with PI (50 µg/ml) and were gated for the Sub G_0_ population as P1 in a Pulse Area Vs Pulse Width of the PE- 561 nm laser. (**c**) PARP-1 activation was monitored with PAR polymer production by western blot analysis at various time points upon AEBP1 down regulation. Un-transfected and scrambled siRNA (ScSi) treated U138MG cells were used as controls. (**d**) FACS analysis of Mitotracker measured at 577 nm in a Count Vs. Pulse-Area graph. Cells with live mitochondria metabolize the chloro-methyl moiety converting to a red fluorescent dye which can be read at 577 nm. The number in the graph is an average of three independent experiments indicating the percentage of cells showing signal at given time point and within the threshold intensity.

Caspase mediated apoptosis is characterized by the fragmentation of genomic DNA generating nucleosomal ladder pattern^15,24^. When we analyzed the genomic DNA of AEBP1 silenced U138MG cells, we did not observe any pattern of DNA fragments reminiscent of nucleosomal ladder (Fig. 2a; lane 6–9). However, we did observe a smear of genomic DNA approximately around 50 Kb in AEBP1 siRNA transfected U138MG cells indicating that DNA is cleaved randomly to generate approximately 50 Kb large sized fragments. However, we observed DNA ladder formation in doxycycline treated U138MG cells at 50 ug/ml (Supplementary Fig. 2). We further corroborated this large scale DNA fragmentation by FACS analysis of cells after staining with propidium iodide. These results shown in Fig. 2b reveal that on 7^th^ day and 9^th^ day post AEBP1 siRNA transfection, most of the cells were in P1 quadrant, representative of sub G_0_/G_1_ cells having DNA content less than ‘n’, which is indicative of large scale fragmentation of DNA. As previously shown, cell proliferation was impeded and viability decreased from 5^th^ day onwards^8^.

Apoptosis inducing factor (AIF) is a mitochondrial flavoprotein involved in bioenergetics and redox metabolism^29–31^. Loss of mitochondrial outer membrane potential causes leakage of AIF^16^. AIF then leaches into the cytosol and further translocates to the nucleus, where it participates in peripheral chromatin condensation and chromatinolysis^32,33^. This release of AIF from mitochondria is believed to be triggered by PARP-1. The synthesis of PARP-1 is linked to DNA repair in the nucleus^17^. Excessive activation of PARP-1 leads to an intrinsic cell death program, poly (ADP-ribose) (PAR) polymer (PAR) that is synthesized in the nucleus and released into the cytoplasm where it triggers the release of AIF from the mitochondria^34,35^. Since PARP-1 was not cleaved under AEBP1 depleted conditions in U138MG cells (Fig. 1a) we probed for PARP-1 activation induced PAR polymer formation. PAR polymer was measured in a time dependent manner upon AEBP1 down regulation and it was observed that there was an increase in the formation of this polymer on the 3^rd^ day which was increased several fold on the 5^th^ day post AEBP1 down regulation (Fig. 2c). However, we did not detect PAR polymer from 7^th^ day onwards. From these observations we inferred that AEBP1 down regulation causes caspase independent cell death and this cell death is mediated by PARP-1 and this might release the AIF from the mitochondria.

### Loss of MOMP and AIF release from the mitochondria

Mitochondria sequester various pro apoptotic proteins within its inter-membrane space; consequently, the integrity of the outer mitochondrial membrane is essential for a healthy normal cell^18,36^. The disruption of the outer mitochondrial membrane is essential for initiating cell death. We therefore examined whether there is a loss of outer mitochondrial membrane potential/loss of membrane integrity in AEBP1 depleted U138MG cells. To investigate this possibility, we used MitoTracker Red CMXRos, a red-fluorescent dye that stains mitochondria in live cells and its accumulation within the mitochondria is dependent on positive membrane potential. The loss of membrane integrity and subsequent mitochondrial leakage causes drop in the membrane potential and hence reduction in staining intensity. As can be seen in Fig. 2d, we observed that 91.17% of the cells treated with scrambled siRNA showed positive MOMP whereas AEBP1 depleted cells showed loss of MOMP as days of treatment progressed. There was a significant decrease in Mitotracker staining on 5^th^ day post transfection (70.66%) which showed a further decrease on 7^th^ day post AEBP1 siRNA (86.66%) and 9^th^ day (86.94%) post AEBP1 siRNA treatment.

**Figure 3:**
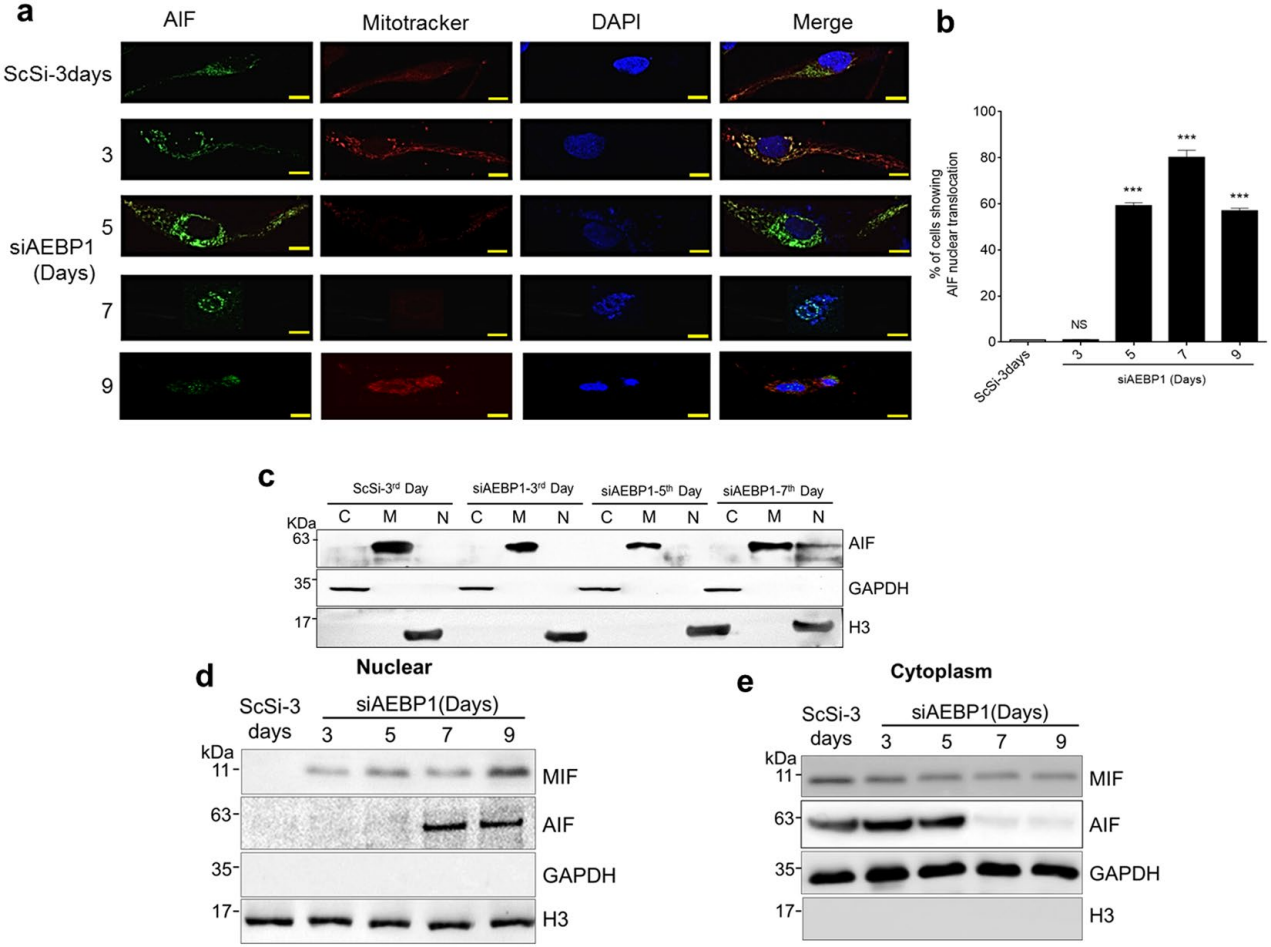
AEBP1 down regulation releases AIF from mitochondria which translocates to the nucleus along with MIF. (**a**) Triple Immunofluorescence images of U138MG cells representing AIF and mitotracker together with DAPI showing movement of AIF from mitochondria to nucleus and loss of MOMP upon AEBP1 silencing in U138MG cells. (Scale Bar-10 μm). (**b**) Graphical representation of percentage of cells showing AIF nuclear localization upon AEBP1 silencing up to 9 days. Cells were counted manually for each day post transfection with siAEBP1. ***p < 0.0001 and NS, Non-significant. (Scrambled (ScSi) versus siAEBP1 treated cells). One-way ANOVA followed by Dunnett’s test was used to evaluate the statistical significance. The data are expressed as mean ± SD (n = 3). (**c**) Subcellular localization of AIF. Upon AEBP1 silencing, cells were subjected to sub cellular fractionation yielding cytoplasm, nuclear extract and membrane rich fractions. Western blotting of different fractions shows AIF retention in membrane rich fractions up to 5^th^ day post siAEBP1 transfection. AIF intensity shows an increase in the nuclear fraction on 7^th^ day post siAEBP1 transfection. Histone H3 and GAPDH were used as nuclear and cytoplasmic fractions marker respectively. (**d**,**e**) Subcellular localization of MIF and AIF in U138MG cells upon AEBP1 silencing. Histone H3 and GAPDH are used as nuclear and cytoplasmic marker.

As a consequence of this loss of mitochondrial outer membrane integrity, we expected AIF to be released from the mitochondria. The release of AIF was monitored by both immunofluorescence and sub cellular fractionation. As can be seen in Fig. 3a,b, we observe the release of AIF from mitochondria from 5^th^ day onwards. AIF tends to accumulate in the peri-nuclear region by the 5^th^ day and ultimately moves into the nucleus on the 7^th^ day and is predominantly in the nucleus on the 9^th^ Day. This was further validated by western blotting analysis of the sub cellular fractions (Fig. 3c). We did not observe any AIF in the nuclear fraction in scrambled siRNA treated U138MG glioma cells. Despite molecular association studies on AIF, yet we still do not understand the molecular function of AIF inside the nucleus particularly in chromatinolysis^15,35^. Recently, Wang *et al*.^19^ have identified macrophage inhibitory factor (MIF) as a PARP-1-dependent AIF associated nuclease that is required for parthanatos and also have shown that AIF is required for recruitment of MIF to the nucleus. An earlier study has shown that MIF is highly expressed in human glioma cell lines which increases further with the grade of malignancy of human glioma patients^37^. We have observed that MIF is expressed in the four selected glioma cells (Supplementary Fig. 3) and in this background, we hypothesized that MIF’s nuclease activity and AIF-mediated recruitment are required for AEBP1 down regulation induced parthanatos. To prove this hypothesis, we checked for the subcellular localization of MIF in AEBP1 down regulated U138MG cells by western analysis. As shown in the Fig. 3d,e, we observed that MIF levels gradually increased in the nuclear fractions of AEBP1 silenced U138MG cells from 3^rd^ to 11^th^ day while there was a corresponding decrease in its cytoplasmic level. These observations confirm that AEBP1 down regulation in U138MG cells leads PARP-1 over-activation resulting in higher PAR polymer formation. It is quite likely that PAR polymers which translocate from nucleus to cytoplasm, trigger the AIF release from mitochondria by altering the MOMP. In the presence of nuclear translocated AIF, MIF more efficiently cleaves the genomic DNA into larger fragments^19^.

### AEBP1 depletion increases γH2AX foci in the nucleus

**Figure 4:**
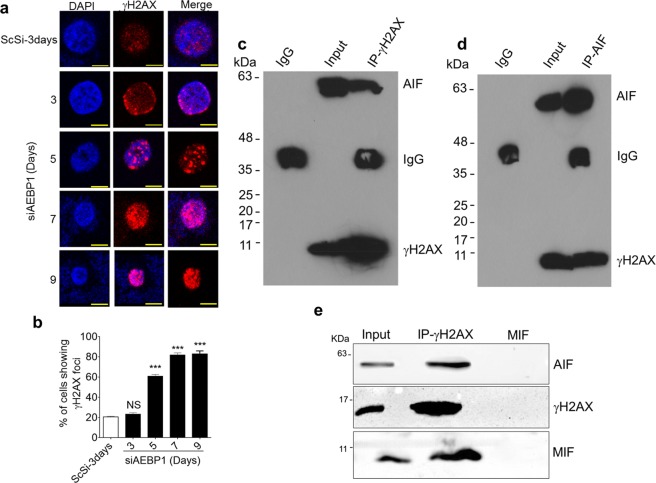
AIF translocation into nucleus results in γH2AX foci accumulation. (**a**,**b**) Immunofluorescence images showing progressive increase in γH2AX foci upon increasing days of AEBP1 down regulation. Fluorescence intensity was quantitated and represented in panel b. (Scale Bar-10 μm) ***p < 0.0001 and NS, Non-significant. (Scrambled (ScSi) versus siAEBP1 treated cells). One-way ANOVA followed by Dunnett’s test was used to evaluate the statistical differences. The data are expressed as mean ± SD (n = 3). (**c**) AIF-associates with γH2AX: Chromatin fraction was isolated from U138MG cells 7 days post siAEBP1 transfection. γH2AX was immunoprecipitated using Protein A agarose beads and the eluate from the matrix was probed with anti-AIF and anti-γH2AX antibodies. (**d**) γH2AX associates with AIF: Chromatin fraction was isolated from the U138MG cells 7 days post siAEBP1 transfection. AIF was immunoprecipitated using Protein A agarose beads and the eluate from the matrix was probed with AIF and γH2AX antibodies. (**e**) γH2AX associates with MIF: Chromatin fraction was isolated from U138MG cells 7 days post siAEBP1 transfection. γH2AX was immunoprecipitated using Protein A agarose beads and the eluate from the matrix was probed with anti-AIF, anti-γH2AX and anti-MIF antibodies.

Since AEBP1 down regulation resulted in MIF mediated large scale DNA fragmentation in the nucleus because of AIF translocation, we predicted that it is quite likely that double strand breaks may be accumulating under AEBP1 depleted condition. We examined the levels of double strand breaks in U138MG cells following AEBP1 down regulation by monitoring γH2AX foci through immunofluorescence analysis. Cancer cells by default tend to accumulate double strand breaks^38^ which are very low in control un-transfected and scrambled siRNA treated U138MG cells as shown in Fig. 4a. However upon AEBP1 down regulation, there is a steady increase in the number of γH2AX foci progressively on 5^th^, 7^th^ and 9^th^ day post AEBP1 siRNA transfection (Fig. 4b). It is also interesting to note that the γH2AX foci are predominantly in the nuclear periphery to begin with and at later time points, there were many more foci accumulating in the core of the nucleus. We were curious to know whether these γH2AX foci are associated with AIF bound chromatin domains in AEBP1 depleted U138MG cells. We isolated chromatin fractions from the 7^th^ day post AEBP1 siRNA transfected cells and immunoprecipitated with either AIF or γH2AX antibodies and probed for the other protein and the results are shown in Fig. 4c,d. We checked whether MIF also associates with the γH2AX-associated chromatin which was demonstrated in Fig. 4e. These results clearly demonstrate that γH2AX foci are in the same chromatin domain interacting with AIF-MIF in the nucleus.

### Increase in DSB foci is mediated by PI3KCB down regulation

**Figure 5:**
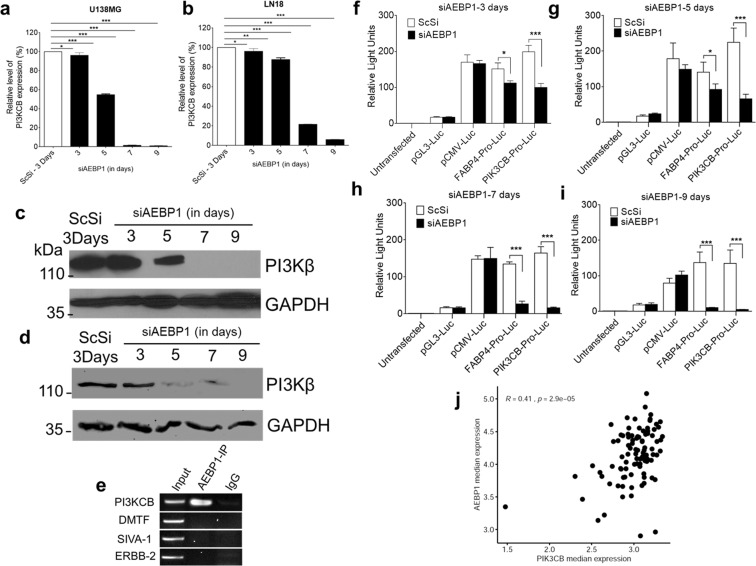
AEBP1 positively regulates PI3KCB. (**a**,**b**) qRT-PCR for PI3KCB expression in ScSi control and siAEBP1 treated U138MG (**a**) and LN18 (**b**) cells. *p < 0.05 and ***p < 0.0001. (Scrambled (ScSi) versus siAEBP1 treated cells). One-way ANOVA followed by Dunnett’s test was used to evaluate the statistical differences. The data are expressed as mean ± SD (n = 3). (**c**,**d**) Western blot analysis of PI3KCB upon AEBP1 down regulation in U138MG (**c**) and LN18 (**d**) cells. The 5^th^ day lane shows decrease in PI3KCB signal which is completely absent in post 7^th^ day of siAEBP1 transfected cells. ScSi treated cells was used as a negative control. (**e**) ChIP-PCR of PI3KCB promoter sequence (−2Kb) showing AEBP1 interaction with the PI3KCB promoter in U138MG cells. DMTF, SIVA-1 and ERBB2 gene promoter loci were used as negative controls. (**f**–**i**) Luciferase reporter assay driven by PI3KCB promoter in U138MG cells upon silencing of AEBP1 for different time periods of time. Cells were transfected with constructs of pGL3-Luciferase Basic vector containing one of the following: CMV promoter, promoter region of the gene FABP4 (transcriptionally repressed by AEBP1), and PI3KCB promoter region. Luciferase assay was performed at different time points after siAEBP1 treatment. *p < 0.05 and ***p < 0.0001. (Scrambled (ScSi) versus siAEBP1 treated cells). One-way ANOVA followed by Dunnett’s test was used to evaluate the statistical significance. The data are expressed as mean ± SD (n = 3). (**j**) Correlation between AEBP1 and PIK3CB expression levels in GBM patients in relation to normal brain tissues retrieved from TCGA database through cBioPortal (http://www.cbioportal.org/). White dots refer to individual GBM patients. r - Pearson correlation coefficient and p – statistical significance.

**Figure 6:**
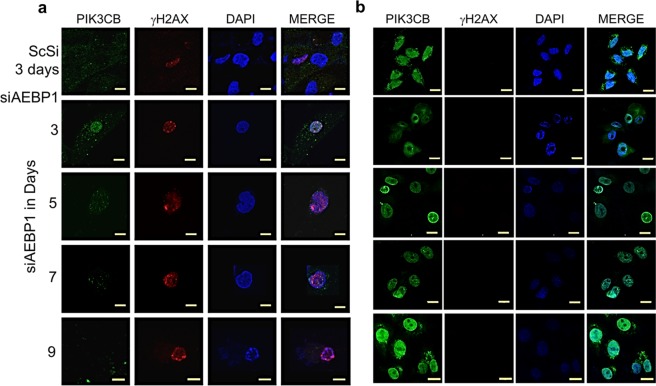
Ectopic expression of PIK3CB impedes the γH2AX foci accumulation in AEBP1 depleted U138MG cells. (**a**) Triple Immunofluorescence images of AEBP1 down regulated cells at different time points showing an increase in γH2AX foci showing translocation of PI3Kβ to the nucleus 3days post siAEBP1 transfection. At both time points, PI3Kβ is progressively down regulated. (Scale Bar −10 μm). (**b**) Overexpression of PIK3CB in trans abrogates accumulation of γH2AX foci in AEBP1 down regulated cells: Immunofluorescence images showing decreased γH2AX foci accumulation upon AEBP1 downregulation under the condition of PI3KCB overexpression. U138MG cells were transfected with pcDNA-PIK3CB ORF clone construct and treated with ScSi and siAEBP1 RNA for different time points. (Scale Bar −10 μm).

Next, we addressed the possible reasons for the accumulation of DSBs and DNA fragmentation in the absence of AEBP1. The basal levels of PAR are very low in cancerous cells^17^. However, excessive activation of PARP-1 leads to 10–500-fold increase in PAR polymer formation^39^. PARP-1 is activated by DNA damage that is inherent to cancer and massive DNA damage may lead to rapid activation of PARP-1 and PAR formation. Thus, an obvious question that arises at this point is the reason for such a significant increase in PAR polymer formation in AEBP1 depleted U138MG cells. Recently it has been shown that PI3Kβ is necessary for the double stranded DNA break sensing^40^. Inhibition of PI3Kβ has been shown to retard the DNA damage repair process^21^ and cause genomic instability. Previously it has been shown that down regulation of PI3KCB leads to loss of cell proliferation, cell cycle arrest and apoptosis^21^. This function for PI3Kβ is independent of its kinase activity^41^. The micro array data from our previous work showed a 1.52 fold down regulation of PI3KCB upon silencing AEBP1^8^. We have reconfirmed that PI3KCB is indeed down regulated under AEBP1 down regulated conditions at mRNA and protein level in glioma cells (Fig. 5a–d). When we started looking for possible reasons for PI3KCB down regulation, we observed that the PI3KCB promoter (−2Kb) has AEBP1 binding motifs. PI3KCB was also listed as one of the genes with AEBP1 binding motif in our previous ChIP-chip analysis^8^. We further validated this by chromatin immune precipitation with anti-AEBP1 antibody and subsequent semi-quantitative and quantitative PCR of the immune precipitated DNA fragments (Fig. 5e; Supplementary Fig. 4). Next, we proceeded to demonstrate that AEBP1 does regulate PI3KCB gene regulation by carrying out luciferase reporter assay with −2000 to +200 bp of PI3kinase 110 CB cloned into pGL3 vector. For this purpose, both U138MG and HeLa cells were transfected with pGL3 construct and other controls along with β-galactosidase construct to normalize for transfection efficiency. We observed PIK3CB promoter was indeed functional in U138MG cells and the promoter activity was decreased upon AEBP1 down regulation (Fig. 5f–i). There was no functional PIK3CB promoter activity in HeLa cells which does not express AEBP1 (Supplementary Fig. 5). Hence, it is very likely that down regulation of PI3KCB is most likely the reason for accumulation of DSB foci observed in our experiments. To identify the positive correlation between AEBP1 and PIK3CB in *in vivo*, we downloaded the GBM TCGA dataset^42^ from cBioportal^43^ and extracted median expression values of AEBP1 and PIK3CB from PTEN homozygously deleted (n = 12) and non-altered PTEN (n = 154) samples. Correlation between the AEBP1 and PIK3CB was computed using cor.test function in R software and plotted scatter plot using ggplot2 R package. As shown in the Fig. 5j, there was strong positive correlation between AEBP1 and PIK3CB in GBM cases compared to normal tissues. To further substantiate our hypothesis that down regulation of PI3KCB is a causative event for DSB accumulation in AEBP1 down regulated cells, we carried out a time course dependent measurement of PI3Kβ disappearance and appearance of γH2AX foci after AEBP1 down regulation by immunofluorescence. As can be seen in Fig. 6a, upon AEBP1 down regulation PI3Kβ moves to the nucleus and PI3K foci can be observed at 3 days post transfection which subsequently decreases significantly on 5^th^, 7^th^ and 9^th^ days. Conversely, γH2AX foci continuously increase from 3^rd^ day to 9^th^ day post transfection. We validated these results by over expressing PI3KCB in U138MG cells (Supplementary Fig. 6) and visualized the γH2AX foci accumulation upon silencing AEBP1. As expected γH2AX foci accumulation was abrogated by PIK3CB over expression (Fig. 6b) when compared to pcDNA3.1-vector alone treated U138MG cells (Supplementary Fig. 7). Thus, there is a reciprocal relationship between down regulation of PI3KCB and the increase in γH2AX foci in AEBP1 depleted cells.

### AEBP1 down regulation induces caspase dependent cell death in PTEN-proficient glioma cells

**Figure 7:**
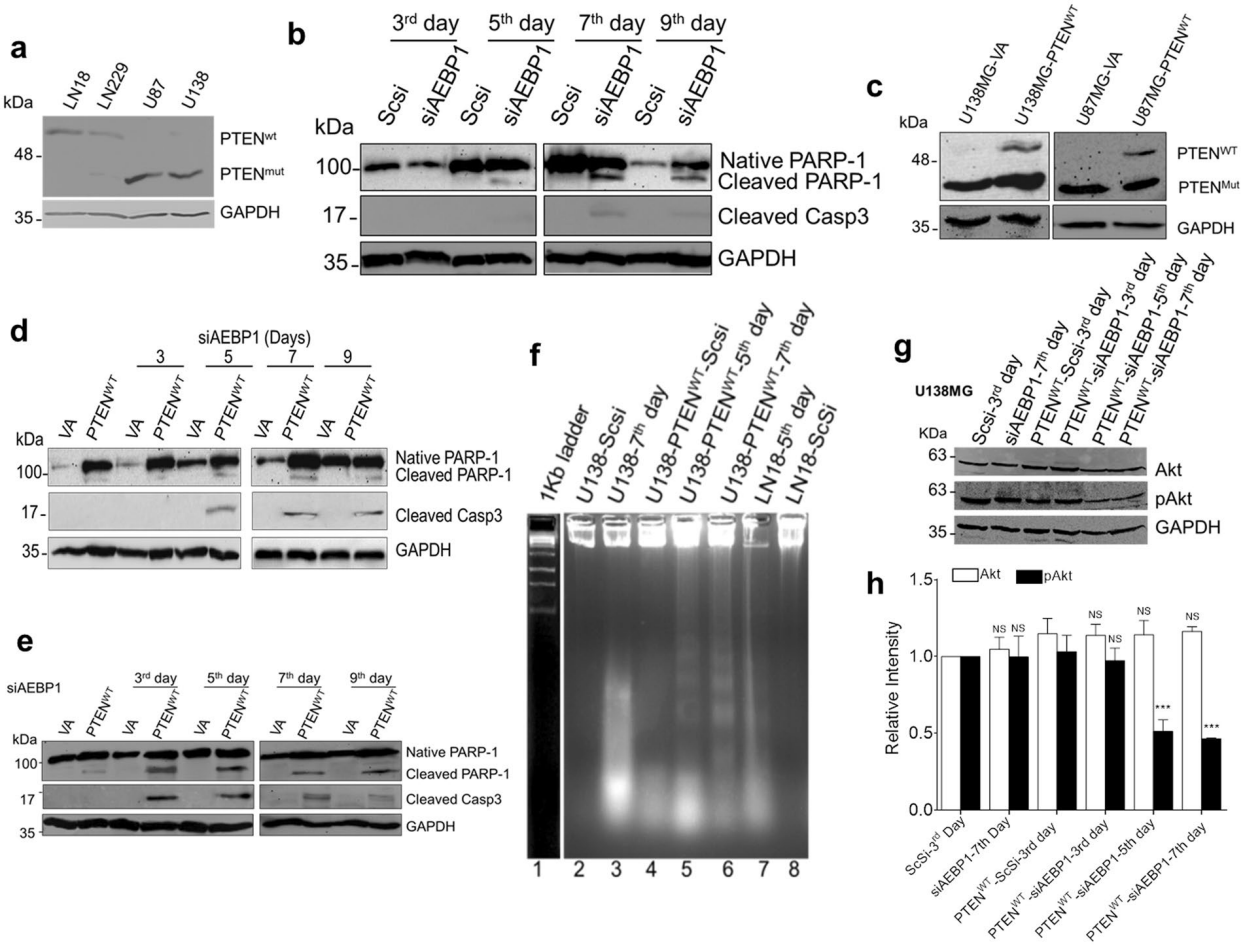
Ectopic expression of PTEN^WT^ (PTEN-wild type) in AEBP1 depleted U138MG and U87MG cells restores the caspase dependent cell death pathway. (**a**) Western blot analysis of wild type and mutant PTEN protein expression in different GBM cell lines. (**b**) Levels of PARP-1, cleaved PARP-1, cleaved caspase 3 upon AEBP1 silencing in LN18 cells at different time points. GAPDH was used as a loading control in all the experiments. (**c**) Western blot analysis of ectopic expression of PTEN^WT^ in U138MG and U87MG cells. U138MG and U87MG cells were transfected with pcDNA 3.1^+^ (VA) or pcDNA-PTEN^WT^ (PTEN^WT^) constructs. Total protein was extracted after 72 hrs of post transfection and probed for PTEN. (**d**,**e**) Levels of PARP-1, cleaved PARP-1, cleaved caspase 3 upon AEBP1 silencing in U138MG (**d**) and U87MG (**e**) cells after PTEN^WT^ transfection at different time points. GAPDH was used as a loading control in all the experiments. (**f**) Accumulation of large scale DNA fragments was observed in U138MG-ScSi and U138MG-PTEN^WT^-ScSi cells on 7^th^ day of post siAEBP1 transfection (lane 3 and 4). Ectopic expression of pcDNA-PTEN^WT^ in U138MG cells induces DNA ladder formation upon AEBP1 silencing on 5^th^ and 7^th^ day of post transfection (lane 5 and 6). AEBP1 silencing in LN18 cells shows DNA ladder formation on 5^th^ day of post transfection (lane 7) compared to ScSi treated cells (lane 8). (**g**) Western blot analysis for Akt and phospho-Akt (pAkt) upon AEBP1 down regulation in pcDNA-PTEN^WT^ cells. (**h**) Densitometric analysis of Akt and pAkt in AEBP1 depleted cells transfected with pcDNA-PTEN^WT^ construct. One-way ANOVA followed by Dunnett’s test was used to evaluate the statistical differences. ***p < 0.0001 and NS, Non-significant. (Scrambled (ScSi) versus siAEBP1 treated cells). The data are expressed as mean ± SD (n = 3).

It is well-known that genetic alterations of tumor suppressor genes could play an essential role in the pathogenesis of glioma. It has been reported that mutation in PTEN are most frequently found in GBM and has been implicated in the pathogenesis of high-grade glioma^44,45^. Furthermore, PTEN mutations were detected in more than 70% of glioma cell lines, and the frequencies of these mutations were significantly higher in cell lines than the glioblastoma tumors^46^. To verify the PTEN status of glioma cells, we probed for PTEN with anti-PTEN antibody in U87MG, U138MG, LN18 and LN229 cells. As shown in the Fig. 7a, PTEN-deficient cells such as U87MG and U138MG cells express mutant PTEN (42KDa) whereas PTEN-proficient cells express wild-type PTEN (55KDa). We also verified this by sequencing the cDNA of U87MG, U138MG, LN18 and LN229 cell lines with two pairs of PTEN wild-type sequence-specific primers (Supplementary Table 2) and found that U87MG and U138MG cells possess two different missense mutations in PTEN exon sequences while there were no mutations in PTEN exon sequences of LN18 and LN229 cells (Supplementary Table 1).

An earlier study has revealed that AEBP1 negatively regulates adipose tissue PTEN levels, in conjunction with its role in proliferation and differentiation of pre-adipocytes^47^. PTEN also acts as a critical determinant of cell fate, regarding senescence and apoptosis in glioma cells under ionization radiation exposure^48^. We were curious to examine whether the process of cell death observed in PTEN deficient glioma cells (U138MG and U87MG) also occurs in PTEN proficient glioma cells (LN18 and LN229) upon AEBP1 down regulation. We performed AEBP1 silencing in LN18 and LN229 cells and observed maximum down regulation at 72 hrs of post-transfection for LN18 and LN229 cells respectively (Supplementary Fig. 8a,b). Thus, siRNA pool was replenished every 60 hours in LN18 and LN229 cells for over a period of 11 days and assessed for cellular proliferation by the MTT assay. As shown in Supplementary Fig. 1c, there was no aberrant change in the cell viability until the 3^rd^ day of post-transfection. Nevertheless, there was a significant reduction in the cell viability from 5^th^ day onwards suggesting that silencing of AEBP1 resulted in cell death in PTEN-proficient cells also. Interestingly, the activation of caspase 3, loss of MOMP and cleavage of PARP-1 was observed after 5 days of post-transfection in LN18 cells upon AEBP1 down regulation (Fig. 7b) in contrast to PTEN-deficient U138MG and U87MG cells. Together, these data show that AEBP1 down regulation induces caspase-dependent cell death in PTEN-proficient glioma cells.

### Ectopic expression of wild type PTEN in U138MG cells induces the caspase and Akt dependent cell death pathway upon AEBP1 down regulation

From these results described above, we conclude that PTEN status in glioma determines the cell fate either through caspase-dependent or independent cell death pathway. Thus, we  were curious to know what happens if we provide wild type PTEN (PTEN^WT^) in trans to PTEN-deficient cells. We transfected the pcDNA-PTEN^WT^ constructs into U138MG and U87MG cells and scored for caspase 3 and PARP-1 activation after silencing AEBP1. PTEN^WT^ expression was observed after 72 hrs of post-transfection when compared to pcDNA 3.1^+^ construct alone transfected cells (Fig. 7c). We observed cleaved caspase 3 and PARP-1 after the 3^rd^ day in U87MG cells and 5^th^ day in U138MG cells of AEBP1 post silencing and increased subsequently on 7^th^ and 9^th^ day on both cells (Fig. 7d,e). These results indicate that PTEN-deficient cells choose caspase dependent cell death when PTEN is provided in trans. We also found that nucleosomal ladder formation in pcDNA-PTEN^WT^-U138MG cells upon AEBP1 down regulation (Fig. 7f). This trend was corroborated with PTEN-proficient cells LN18 on 5^th^ day of post AEBP1 down regulation (Fig. 7b). These results show that AEBP1 depletion induces caspase-dependent and PARP-1 mediated cell death in the presence of PTEN^WT^ in the PTEN-deficient cells.

It is well-known that Phosphatidylinositol-3-kinase (PI3K) pathway, a downstream target pathway plays an essential role in regulating cell death and cell cycle arrest^49,50^. PTEN induces apoptosis and cell cycle arrest through PI3K/Akt dependent and independent pathways in breast cancer cells^51^. Importantly, correction of PTEN mutations in glioblastoma cell lines through gene editing resulted in reduced cell proliferation which was Akt-dependent in 42MGBA cells and Akt-independent in T98G cells^52^. To assess the effect of ectopic expression of PTEN^WT^ in U138MG cells we performed western blots on protein lysates using Akt specific and pAkt specific antibodies. We observed that expression level of Akt was unaltered in all conditions but there was a significant decrease in pAkt levels from the 3^rd^ day of AEBP1 silencing under PTEN^WT^ ectopic expression conditions (Fig. 7g,h; Supplementary Fig. 9). We also validated this trend in PTEN-proficient cells LN18 and LN229 upon down regulation of AEBP1 (Supplementary Fig. 10). These results show that ectopic expression of PTEN^WT^ in U138MG cells induces the Akt dependent cell death pathway in AEBP1 depleted cells. Having these results, we were interested to see the correlation between AEBP1 and PIK3CB expression in GBM patients under PTEN mutated conditions. We again screened the GBM-TCGA datasets and extracted the data from PTEN deleted GBM tissue samples. As shown in the Supplementary Fig. 11 there is no significant correlation between AEBP1 and PI3Kbeta expression in PTEN deleted GBM cases. This shows that PI3Kbeta expression in clinical GBM tumor tissues was independent of PTEN status. Overall, these results show that PTEN status determines the choice of cell death pathway in AEBP1 depleted glioma cells.

## Discussion

Despite the enormous progress in our knowledge on the molecular and genetic basis of glioma, little progress has been made on molecular de-regulation of cell death pathways in glioma^53^. Glioblastoma multiforme (GBM) is the most malignant tumor of the brain, associated with one of the worst survival rates among all human cancers^54^. Even after aggressive multimodal treatment of surgical resection, local radiotherapy, and systemic chemotherapy, the median survival after diagnosis is still around 12–15 months^55^. After the advent of next-generation sequencing technology, it is clear that glioblastoma exhibits aberrant metabolic reprogramming^56^ and striking cellular and molecular heterogeneity^9^ which prevents the effectiveness of the new clinical practice.

In an effort to identify glioblastoma specific diagnostic and prognostic markers through microarray analysis, AEBP1 was found up-regulated to greater than 4-fold in primary glioblastoma^1^. AEBP1 is a member of the carboxypeptidase family and is shown to regulate adipogenesis acting as a transcriptional repressor^2^. Transcription factors play a crucial role in the regulation of cellular survival, unrestrained growth and metastatic behavior of all human cancer. In our earlier report, we showed that AEBP1 down regulation results in cell death in both U87MG and U138MG glioma cells^8^. We observed perturbation of several apoptotic and growth associated genes under AEBP1 down regulation. With this background, we were interested to identify the detailed molecular mechanism of cell death in glioma cells under AEBP1 depleted conditions.

In this study, we have shown the role of AEBP1 in initiating caspase-independent cell death in U138MG cells. We observed the disruption of the MOMP and the release of AIF into the cytosol and its subsequent translocation into the nucleus upon silencing AEBP1. One of the immediate effects of AIF translocation into the nucleus is peripheral chromatin condensation^57^. AIF as a mitochondrial oxidoreductase that mediates the caspase-independent cell death^12,58^. AIF is believed to bind directly to DNA^31^ and the crystal structure of human AIF has revealed the presence of a strong positive electrostatic potential at the AIF surface, and this electrostatic interaction between AIF and DNA is independent of the DNA sequence^59^. The mechanism by which AIF triggers DNA fragmentation is still not clear. In *Caenorhabditis elegans*, WAH1, a homolog of AIF, associates with CPS-6, the homolog of mammalian endonuclease G. This association enhances the nuclease activity of CPS-6 and results in apoptotic DNA degradation^13^. It is quite probable that AIF and cyclophilin A collaborate in chromatinolysis^35^. The mechanism of loss of MOMP following AEBP1 down regulation resulting in the release of AIF involves the death effector molecule PARP-1. It is an abundant nuclear protein that acts as a molecular sensor of DNA strand breaks and mediates its repair^32^. Thus, the extent of activation of PARP-1 might be a critical factor that regulates whether cells either die or survive following DNA damage. We observed that a rapid and substantial PAR polymer formation takes place by 5^th^ day post transfection of AEBP1 siRNA. Depending on the stimuli, PAR formed can vary in length and in the frequency of branching. This structural heterogeneity by PAR may be in part responsible for distinguishing between the life and death functions of PARP-1^17^. PAR polymer formed is highly charged in nature and it could conceivably depolarize mitochondria leading to permeability transition and subsequent AIF release. Alternatively, PAR polymer could bind to PAR polymer binding proteins in mitochondria^17,18^, which then triggers AIF release from the mitochondria. At the molecular level PARP-1 activation leads to the accumulation of PAR and the depletion of NAD+ and ATP which might disrupt the MOMP^60^. A very recent study has shown that this bioenergetics collapse might not involve NAD but may be due to PAR-dependent inhibition of glycolysis through a direct inhibition of hexokinase activity^61^. Thus, PAR seems to be a crucial molecule in caspase-independent AIF mediated cell death. Subsequent release of AIF is a two-step process involving cleavage of AIF to be released from the inner membrane and then through the outer mitochondrial membrane by disruption of MOMP^31^. Interestingly, an earlier study has shown that AIF interacts with MIF and recruits MIF to the nucleus and induces chromatinolysis^19^. Furthermore, MIF was upregulated in gastric cancer, pancreatic cancer, melanoma, hepatocellular carcinoma, malignant glioma and cervical adenocarcinoma^62^. It plays an important pathogenetic role in malignant progression of GBM and other CNS tumours^63,64^. In particular, a substantial increase of MIF expression in human GBM has been reported^65,66^. We also observed that MIF was recruited into nucleus along with AIF upon AEBP1 silencing in U138MG cells. Importantly, MIF was identified as a PARP-1-dependent AIF-associated nuclease that is required for parthanatos^19^.

In our previous study we observed enrichment of genes pertaining to activation of PI3Kinase signaling upon AEBP1 down regulation in U87MG and U138MG cells^8^. The present study has also shown that the process of cell death initiation in U138MG glioma cells under AEBP1 depleted condition is possibly mediated through the transcriptional control of PI3KCB. We have demonstrated that AEBP1 binds to the promoter of PI3kinase β resulting in the activation of its transcription. PI3Kinases are a family of enzymes that act downstream of cell surface receptors leading to activation of multiple signaling pathways regulating cellular growth, proliferation, motility, and survival^67^. Disruption of normal PI3K signaling is observed in cancer and many other diseases^22^. Out of the many isoforms of PI3K, PIK3CB from class 1A, also called p110β, has been implicated in tumorigenesis of PTEN-null mouse models and cell lines^21,41^. In human, mutant p110α is oncogenic, whereas p110-β wild-type form is oncogenic^68^. PIK3CB down-regulation results in cell death pathway inactivation and inhibition of growth in both cell-based assays and *in vivo* animal models. This vital function of PIK3CB in PTEN-deficient cancer cells requires its lipid kinase activity suggesting that PTEN-deficient tumors are dependent on p110β signaling^69^. *PTEN* is one of the most frequently mutated tumor suppressor gene in glioma compared to other genes such as *p53*, *p16* and *p14*^ARF^^46^. A complete loss of PTEN has been observed in glioblastoma and endometrial cancer, and PTEN mutations are also associated with advanced cancers and metastases^70^. This may be as a result of the accumulation of PIP3 which promotes the recruitment of the serine/threonine kinase Akt. Akt activation promotes cell survival, proliferation, growth, angiogenesis, and therefore is an important survival signal^70^. Also, PTEN-deficient cells enter senescence following ionization radiation (IR) exposure due to its active Akt signaling whereas PTEN-proficient cells undergo apoptosis upon IR exposure^48^. It is well known that AEBP1 interacts with PTEN and modulates the adiposity and progression of Alzheimer’s disease^47,71^. Independent of well-established Akt kinase activity in cell survival, PI3Kβ is also necessary for sensing double-stranded DNA breaks, as it regulates binding of the Nbs1 sensor protein to Double Strand Breaks (DSBs). In PI3Kβ-deficient cells, the sensing complex MRE-11-Nbs-1 complex fails to recognize DSBs resulting in general defects of ATM and ATR repair pathway and genomic instability^61^. In the present study, we have shown that upon AEBP1 down regulation in PTEN-deficient cells there is a decrease in PI3Kβ which in return can influence ATM and ATR repair pathway and DNA damage. Cancer cells inherently have compromised genomic integrity^38^. Thus, the down regulation of PI3KCB under the AEBP1 silenced conditions results in an accumulation of DSBs as demonstrated by γH2AX foci.

The results presented here also show that AEBP1 down regulation in PTEN-proficient cells (LN18 and LN229 cells) activates caspase-dependent cell death pathway in contrast to caspase independent pathway in PTEN-deficient cells. Previous studies have shown that PTEN gene transfer in glioma cells^72^ or correction of *PTEN* gene via genome editing^52^ suppressed the growth of glioma cells. Most interestingly, ectopic expression of wild type PTEN in both U87MG and U138MG cells induced caspase and Akt dependent cell death mechanism. Thus, PTEN acts as an essential determinant of the process of cell death switch between chromatinolysis and apoptosis in the glioma cell lines U87MG, U138MG, LN18 and LN229 cells. There is evidence that AEBP1 and PTEN interact in the context of adipogenesis and Alzheimer disease^47,71^. It is quite likely that such an interaction might also occur in GBM tumor cells. However, it cannot be ascertained at present how such an interaction might influence the choice of cell death pathway between classical caspase dependent apoptosis and parthanatos. The fact that AEBP1 is necessary for cellular viability in both PTEN-proficient and PTEN-deficient tumor cells, suggests that AEBP1 can be a good potential target for therapeutic intervention in glioma patients in general irrespective of the PTEN status. Therapeutic applications of small molecules targeted at transcription factors are increasingly gaining importance, and it is expected that these approaches may revolutionize the anticancer drug options in the near future.

## Supplementary information

Supplementary table

Data set 1
