# Supplementary material for: AEBP1 down regulation induced cell death pathway depends on PTEN status of glioma cells
Source: Sci Rep. 2019 Oct 10;9:14577. doi: 10.1038/s41598-019-51068-1 (PMC6787275; doi:10.1038/s41598-019-51068-1)
Supplement: Supplementary file 1 — Supplementary table [file 41598_2019_51068_MOESM1_ESM.docx]

**Supplementary Tables**

**AEBP1 down regulation induced cell death pathway depends on PTEN status of glioma cells**

Swati Sinha^1, $^, Arun Renganathan^1, 2, $^, Prathima B. Nagendra^1, 3^, Vasudeva Bhat^1, 4^, Brian Steve Mathew^1^ and Manchanahalli R Satyanarayana Rao^1, *^

^1^ Molecular Biology and Genetics Unit, Jawaharlal Nehru Centre for Advance Scientific Research, Bangalore, Karnataka, India-560064.

^2^ Department of Surgery, Washington University in St. Louis, MO, USA

^3^ Gynaecology Oncology Group, School of Biomedical Sciences and Pharmacy, University of Newcastle, Callaghan, New South Wales, Australia

^4^ Department of Immunology, Faculty of Health Sciences, University of Manitoba, Winnipeg, Manitoba, Canada

$ - These authors contributed equally to this work.

* Corresponding Author

Prof. M.R.S. Rao
Honorary Professor
SERB-YOS Chair Professor
Jawaharlal Nehru Centre for Advanced Scientific Research (Deemed University)
Jakkur, Bangalore - 560 064, India

Email:   [mrsrao@jncasr.ac.in](mailto:mrsrao@jncasr.ac.in)

**Supplementary Tables**

**Supplementary Table 1.**

Localization of PTEN mutations in glioma cell lines. Sequencing of PTEN cDNA of LN18 and LN229 shows no mutations and U87MG and U138MG cells shows two variations.

| **Cell line** | **Codon** | **Amino acid changes and mutation** |
| --- | --- | --- |
| LN18 | WT | None |
| LN229 | WT | None |
| U87MG | 139 (Exon 2)  589 (Exon 5) | AGG(Arg)→GGG(Gly)  AAG(Lys)→GAG(Glu) |
| U138MG | 440 (Exon 5)  782 (Exon 7) | AAG(Lys)→AGG(Arg)  CAG(Gln)→CTG(Leu) |

**Supplementary Table 2.**

**siRNA for AEBP1 and the list of primers used in this study**

| **Gene** | | **siRNA** | **Sequence** |
| --- | --- | --- | --- |
| ON-TARGETplus Human AEBP1 (165) siRNA – SMARTpool- L-009694-00-0050 | | **siRNA-1** | 5’-GGUGGUGGCUCGUUUCAUC-3’ |
|  |  | **siRNA-2** | 5’-GCGAGCGGCUAGUAUCCUA-3’ |
|  |  | **siRNA-3** | 5’-CACCUAAGGCCACCAAGAA-3’ |
|  |  | **siRNA-4** | 5’-UCACAACAGUAGAGACCUA-3’ |
| **Luciferase reporter assay for PI3kinase (110) catalytic sub unit beta (PI3KCB)** | | | |
| **PI3KCB Luc** | FP | 5’ccctcgaggaagtatttaacttttttctctg 3’ | |
|  | RP | 5’ cccaagccataagaaatta 3’ | |
| **ChIP-PCR** | | | |
| **PI3KCB** | FP | 5’ CATATATATAATTTGTGAA 3’ | |
|  | RP | 5’ CATAGAAATTATACCTATT 3’ | |
| **DMTF1** | FP | 5' TTCCCTCCGTTCCACAC 3' | |
|  | RP | 5' CCTGGGTTGGAATGTTTG 3' | |
| **SIVA-1** | FP | 5' CTCCCACCCTTCGTCG 3' | |
|  | RP | 5' GGTGGGACTCGTGCGT 3' | |
| **ERBB2IP** | FP | 5' GGCGAAGGAGAACAAGC 3' | |
|  | RP | 5' CACCGCTTCCTCTCCG 3' | |
| **PTEN^WT^ amplification with *HindIII* and *BamHI* restriction site** | | | |
| **PTEN^WT^-FP** | FP | 5' GCGAAGCTTATGACAGCCATCATCAAAGAGA 3' | |
| **PTEN^WT^-RP** | RP | 5' GCAGGATCCTCAGACTTTTGTAATTTGTGTATG 3' | |
| **PTEN sequencing specific primers** | | | |
| **PTEN^WT^-1** | FP | TATAGGGAGACCCAAGCTGG | |
|  | RP | TCTTGTGAAACAACAGTGCCA | |
| **PTEN^WT^-2** | FP | AGTAACTATTCCCAGTCAGAGG | |
|  | RP | TGGCAACTAGAAGGCACAGT | |
| **Gene Expression Primers** | | | |
| **AEBP1** | FP | 5'-ATGGACTATTACTTTGGGCC-3' | |
|  | RP | 5'-GGGTAGTCCTCCTGGTGTCC-3' | |
| **CAMTA1** | FP | 5'-AATGAGGGAGCGCCTTC-3' | |
|  | RP | 5'-GAACCCGAAAGAACTGTC-3' | |
| **MDM2** | FP | 5'-AACAGCGACACGGAGATC-3' | |
|  | RP | 5'-GCCGTTGCGCTATGTTTG-3' | |
| **UBE3A** | FP | 5'-TACTGTAGATCAACCTGATG-3' | |
|  | RP | 5'-CTTGAGAACTGAAGAGCAC-3' | |
| **ARNT** | FP | 5'-GCAGGACAAAGGAAGAGC-3' | |
|  | RP | 5'-CTCCTTTAGATCGCAGCC-3' | |
| **CD93** | FP | 5'-AACTGTGAGGAGGGAAGG-3' | |
|  | RP | 5'-CCAGGTATTTCGAGTTGTC-3' | |
| **MAPK13** | FP | 5'-AGACGCCGAGATGACTG-3' | |
|  | RP | 5'-GAATCATCAAACGGCTGC-3' | |
| **GAPDH** | FP | 5'-AACAGCGACACCCACTCCTC-3' | |
|  | RP | 5'-GGAGGGGAGATTCAGTGTGGT-3' | |
