# Supplementary material for: AEBP1 down regulation induced cell death pathway depends on PTEN status of glioma cells
Source: Sci Rep. 2019 Oct 10;9:14577. doi: 10.1038/s41598-019-51068-1 (PMC6787275; doi:10.1038/s41598-019-51068-1)
Supplement: Supplementary file 2 — Data set 1 [file 41598_2019_51068_MOESM2_ESM.docx]

**Supplementary Figures**

**AEBP1 down regulation induced cell death pathway depends on PTEN status of glioma cells**

Swati Sinha^1, $^, Arun Renganathan^1, 2, $^, Prathima B. Nagendra^1, 3^, Vasudeva Bhat^1, 4^, Brian Steve Mathew^1^ and Manchanahalli R Satyanarayana Rao^1, *^

^1^ Molecular Biology and Genetics Unit, Jawaharlal Nehru Centre for Advance Scientific Research, Bangalore, Karnataka, India-560064.

#### ^2^ Department of Surgery, Washington University in St. Louis, MO, USA

^3^ Gynaecology Oncology Group, School of Biomedical Sciences and Pharmacy, University of Newcastle, Callaghan, New South Wales, Australia

^4^ Department of Immunology, Faculty of Health Sciences, University of Manitoba, Winnipeg, Manitoba, Canada

$ - These authors contributed equally to this work.

* Corresponding Author

Prof. M.R.S. Rao
Honorary Professor
SERB-YOS Chair Professor
Jawaharlal Nehru Centre for Advanced Scientific Research (Deemed University)
Jakkur, Bangalore - 560 064, India

Email:   [mrsrao@jncasr.ac.in](mailto:mrsrao@jncasr.ac.in)

**Supplementary Figure. 1.**

**Effects of AEBP1 silencing on on-target and off-target genes expression level in U138MG cells**

**a**. qRT-PCR for AEBP1 in ScSi control and siAEBP1 transfected U138MG cells. **b-k.** qRT-PCR for on-target (up-regulated and down-regulated) and off-target genes in ScSi control and siAEBP1 transfected U138MG cells. The values are expressed as mean ± S.D. of three independent experiments. **p < 0.001, ***p < 0.0001 and NS – Non-significant, Scrambled (ScSi) versus siAEBP1 treated cells.


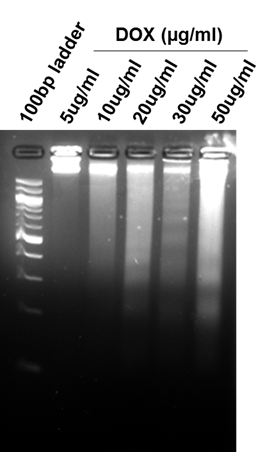


**Supplementary Figure. 2.**

DNA ladder formation in U138MG cells exposed to different concentrations of Doxycycline (5-50µg/ml). Representative image from three independent experiments.


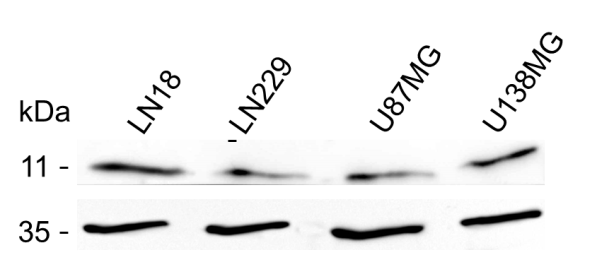


**Supplementary Figure. 3.**

Western blot for MIF protein expression in glioma cell lines. GAPDH was used as a loading Control.

**Supplementary Figure. 4.**

ChIP-qPCR validation of the loci PI3KCB of AEBP1 IP fractions compared with input fractions. Student t test was used to evaluate the statistical differences between Scrambled (ScSi) and siAEBP1 treated cells. The data are expressed as mean ± SD (n = 3), ***p<0.0001

**Supplementary Figure. 5.**

Luciferase promoter assay of the gene PI3KCB in HeLa cells, which is AEBP1 negative cell line. Cells were transfected with constructs of pGL3-Basic vector containing one of the following: CMV promoter, promoter region of the gene FABP4 (transcriptionally repressed by AEBP1), and PI3KCB promoter region. One-way ANOVA followed by Dunnett’s test was used to evaluate the statistical differences between Scrambled (ScSi) versus siAEBP1 treated cells. The data are expressed as mean ± SD (n = 3), ***p<0.0001.

PIK3Cbeta

GAPDH


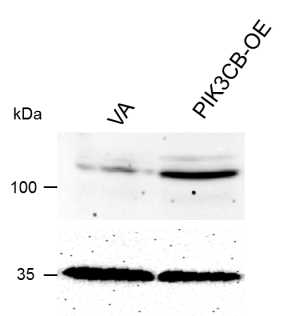


**Supplementary Figure. 6.**

Western blot shows overexpression of PIK3CB in U138MG cells. U138MG cells were transfected with pcDNA 3.1^+^ (VA) or pcDNA-PIK3CB-ORF constructs and total protein was extracted after 72hrs of post transfection and probed for PIK3Cbeta.

**
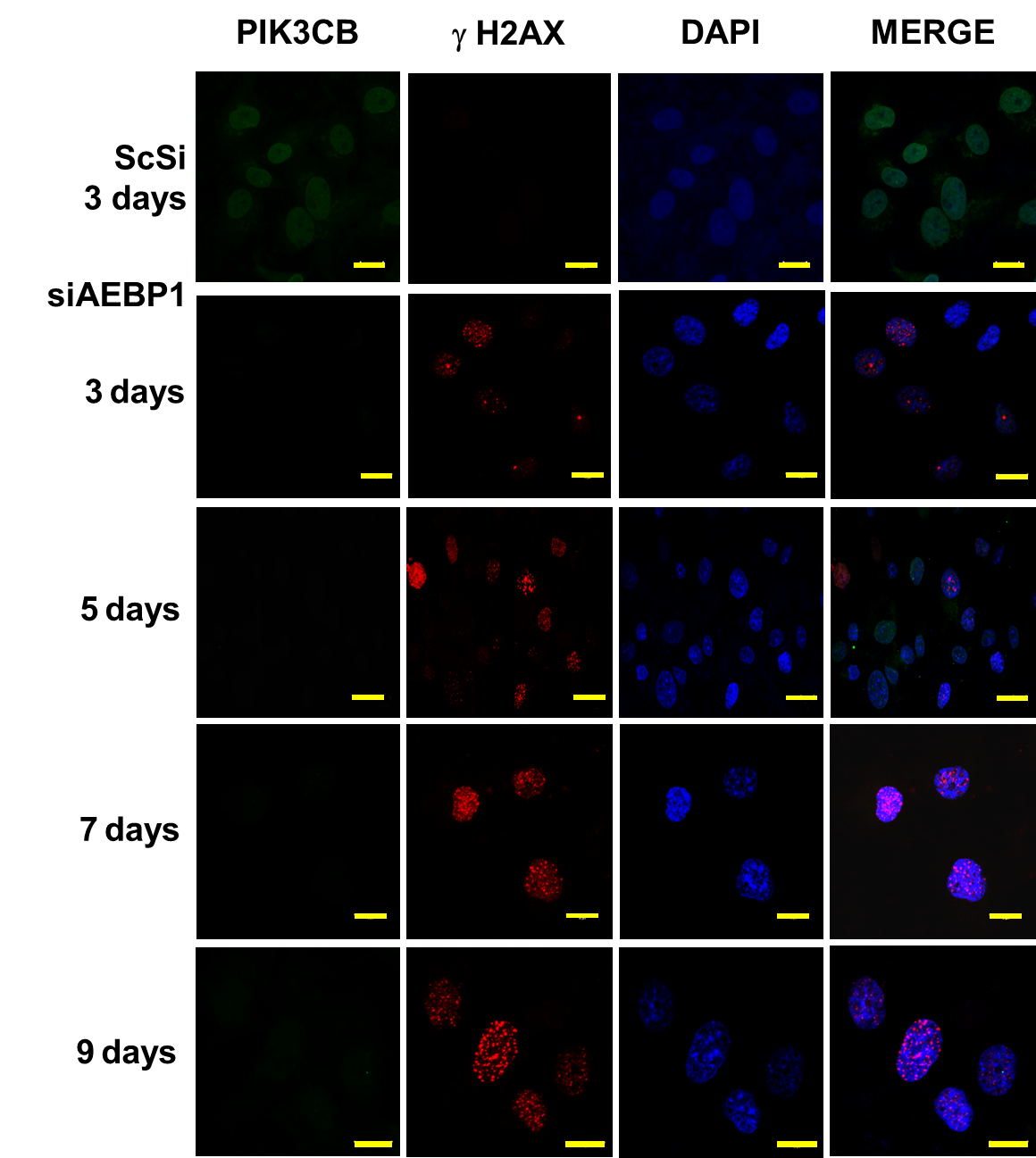
**

**Supplementary Figure. 7.**

Immunofluorescence images showing γH2AX foci accumulation upon AEBP1 downregulation in U138MG cells transfected with pcDNA 3.1^+^ vector and treated with either ScSi or siAEBP1 for different time points as mentioned. Scale Bar - 10μm.

**c**

**b**

**a**

**Supplementary Figure. 8.**

**AEBP1 silencing in LN18 and LN229 cells.**

**a and b**. qRT-PCR for AEBP1 in ScSi control and siAEBP1 transfected LN18 and LN229 cell lines. Maximum downregulation was observed after 72 hours post transfection in both the cell lines, respectively. Hence, siRNA pool was replenished every 60 hours in both cell lines for over a period of 7days and qRT-PCR was performed for AEBP1 gene expression. The values are expressed as mean ± S.D. of three independent experiments. *p < 0.05, **p < 0.001, ***p < 0.001 and ****p < 0.0001, Scrambled (ScSi) versus *si*AEBP1 treated cells.

**(c)** Cell proliferation assay of LN18 and LN229 cells upon AEBP1 silencing after different post transfection time points.


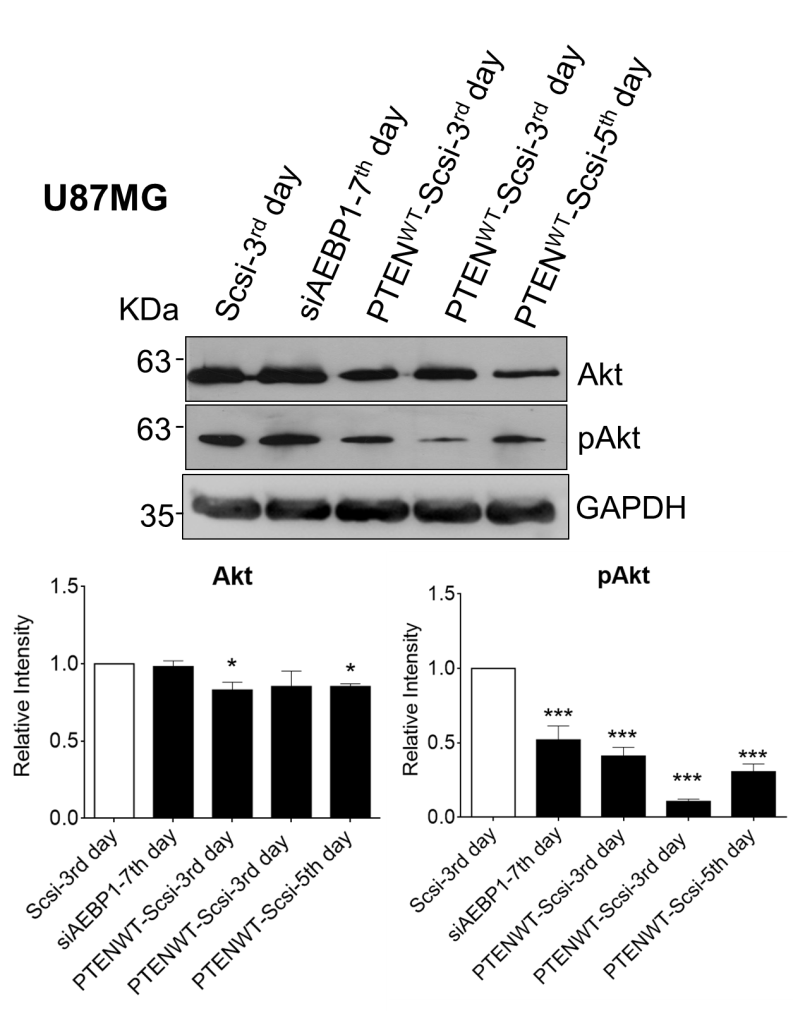


**Supplementary Figure. 9.**

Western blot and densitometry analysis of Akt and phospho-Akt (pAkt) upon AEBP1 down-regulation in pcDNA-PTEN^WT^-U138MG cells. One-way ANOVA followed by Dunnett’s test was used to evaluate the statistical differences. *p<0.05 and ***p < 0.001. (Scrambled (ScSi) versus siAEBP1 treated cells). The data are expressed as mean ± SD (n = 3).


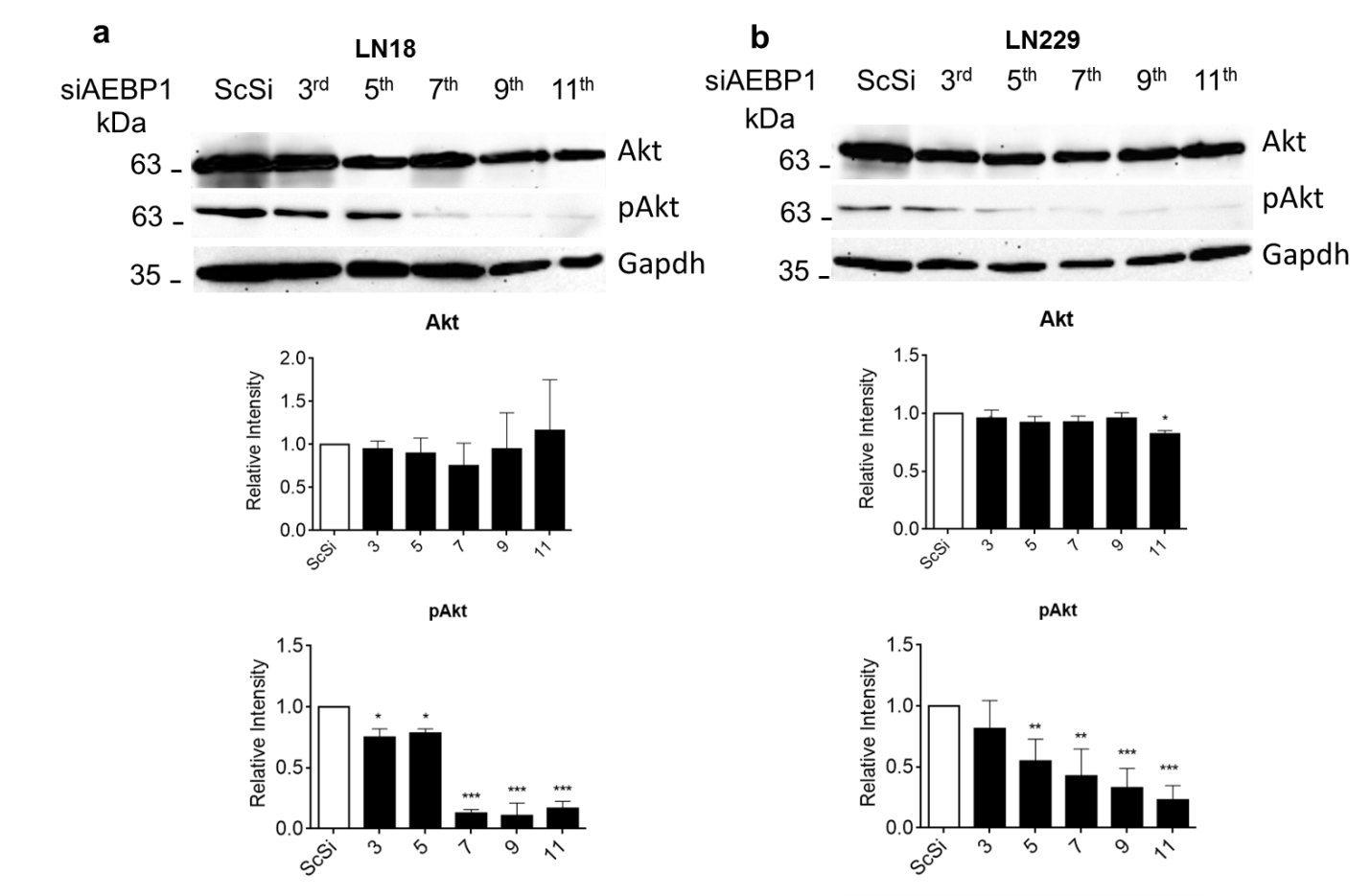


**B**

**A**

**Supplementary Figure. 10.**

Western blot analysis of Akt and pAkt upon Aebp1 down regulation in LN18 (A) and LN229 (B) cells. The values are expressed as mean ± S.D. of three independent experiments. *p < 0.05, **p < 0.01, ***p < 0.001, Scrambled (ScSi) versus *si*AEBP1 treated cells.


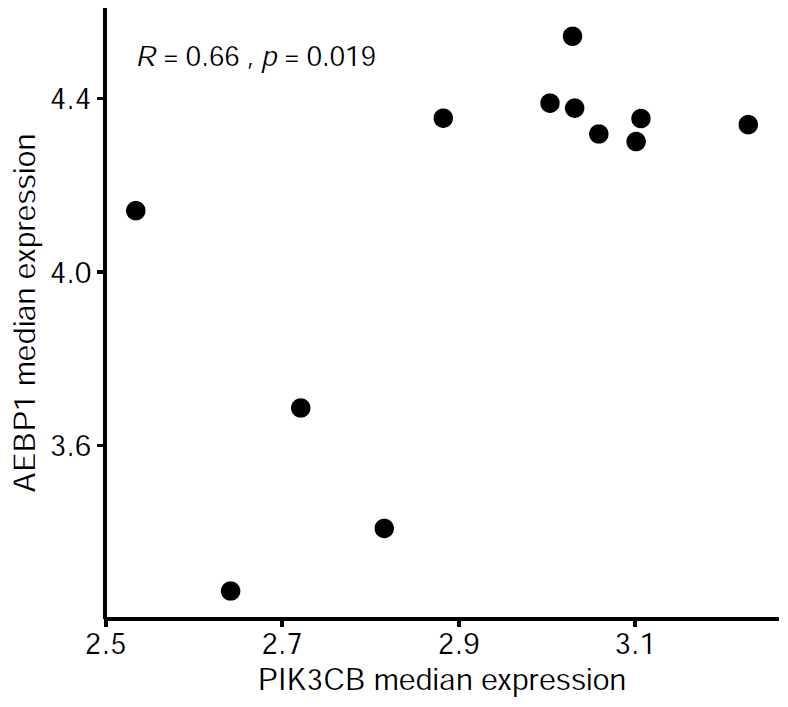


**Supplementary Figure. 11.**

Correlation between AEBP1 and PIK3CB expression levels in PTEN deleted GBM tissues (n=12) from TCGA database through cBioPortal (<http://www.cbioportal.org/>). R-Pearson correlation coefficient and p-P-value.
